# Supplementary material for: Administration of anti-HIV-1 broadly neutralizing monoclonal antibodies with increased affinity to Fcγ receptors during acute SHIVAD8-EO infection
Source: Nat Commun. 2024 Aug 29;15:7461. doi: 10.1038/s41467-024-51848-y (PMC11358508; doi:10.1038/s41467-024-51848-y)
Supplement: Supplementary file 1 — Supplementary Information [file 41467_2024_51848_MOESM1_ESM.pdf]

Supplementary Materials for:

Administration of anti-HIV-1 broadly neutralizing monoclonal antibodies with increased affinity to Fcγ receptors during acute $SHIV_{AD8-EO}$ infection

Joana Dias[1], Giulia Fabozzi[2], Slim Fourati[3], Xuejun Chen[4], Cuiping Liu[4], David R. Ambrozak[1], Amy Ransier[5], Farida Laboune[5], Jianfei Hu[5], Wei Shi[4], Kylie March[2], Anna A. Maximova[4], Stephen D. Schmidt[6], Jakob Samsel[1,7], Chloe A. Talana[4], Keenan Ernste[4], Sung Hee Ko[8], Margaret E. Lucas[8], Pierce E. Radecki[8], Kristin L. Boswell[1], Yoshiaki Nishimura[9], John-Paul Todd[10], Malcolm A. Martin[9], Constantinos Petrovas[2], Eli A. Boritz[8], Nicole A. Doria-Rose[6], Daniel C. Douek[5], Rafick-Pierre Sékaly[3], Jeffrey D. Lifson[11], Mangaiarkarasi Asokan[4], Lucio Gama[1], John R. Mascola[12], Amarendra Pegu[4], and Richard A. Koup[1*]

*Corresponding author:

Richard A. Koup, MD

40 Convent Drive

Vaccine Research Center

National Institutes of Health

Bethesda, MD 20892, USA

Tel: +1 301-594-8585

Email: rkoup@mail.nih.gov

SUPPLEMENTARY FIGURES

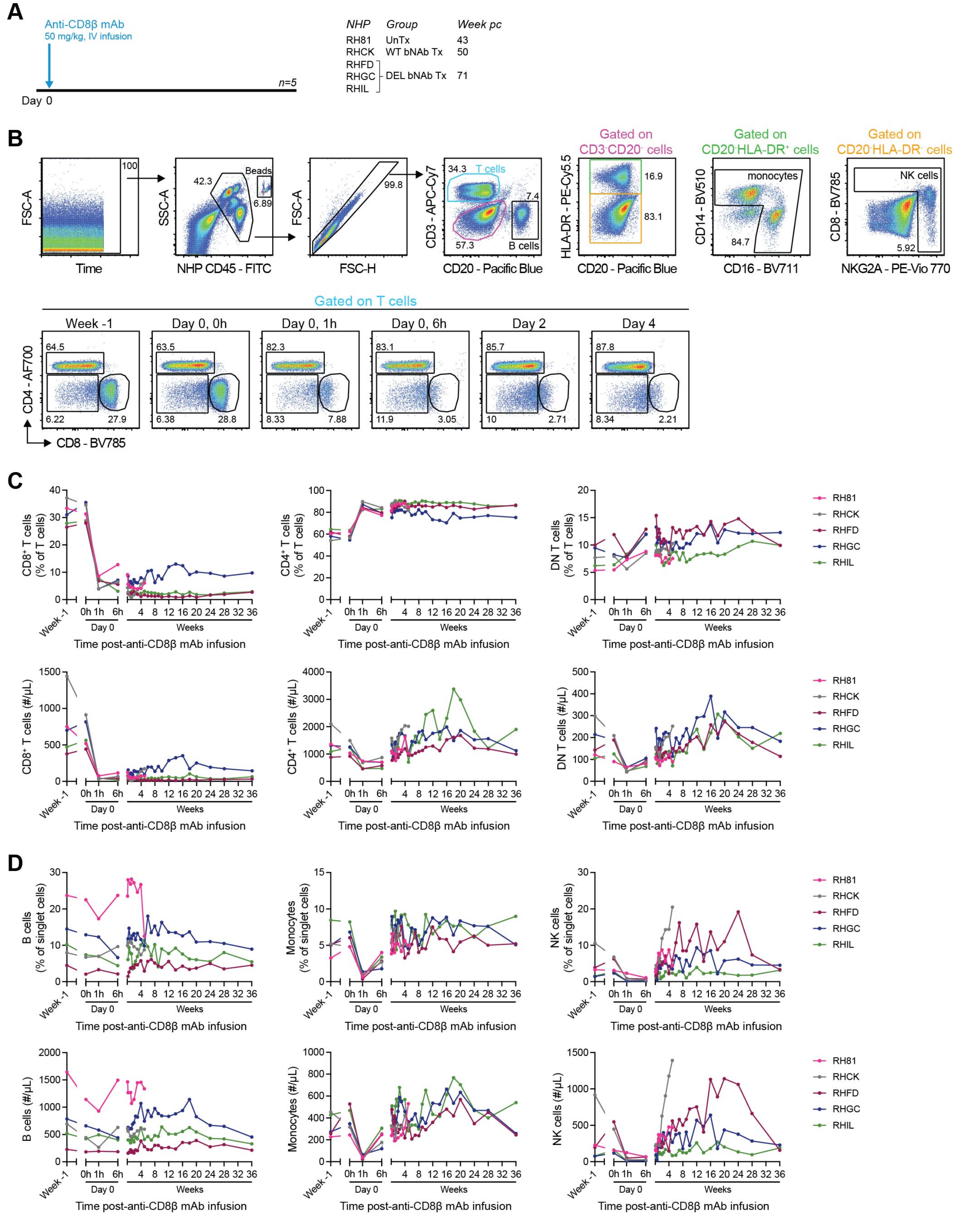

Supplementary Figure 1. CD8^{+} T cell depletion in uninfected monkeys. (**A**) Five SHIV$_{AD8\text{-}EO}$-challenged monkeys that did not develop plasma viremia for more than 30 weeks post-challenge

were infused intravenously with an anti-CD8β mAb at 50 mg/kg. These monkeys came from the untreated group (n=1), WT bNAb-treated group (n=1), and DEL bNAb-treated group (n=3), and anti-CD8β mAb infusion occurred at week 43, 50, and 71 post-challenge, respectively. (**B**) Flow cytometry gating strategy for absolute count of $CD8^+$, $CD4^+$, and DN T cells, B cells, monocytes, and NK cells in whole blood from monkeys infused intravenously with an anti-CD8β mAb. (**C, D**) Frequency (top) and absolute count (bottom) of circulating $CD8^+$ (left), $CD4^+$ (middle), and DN (right) T cells (C), B cells (left), monocytes (middle), and NK cells (right) (D), up to 36 weeks post-anti-CD8β mAb infusion. Each line includes data from one monkey (C, D). Source data are provided in the Source Data file. DN, double-negative; IV, intravenous; NHP, non-human primate; pc, post-challenge; Tx, treatment; UnTx, untreated.

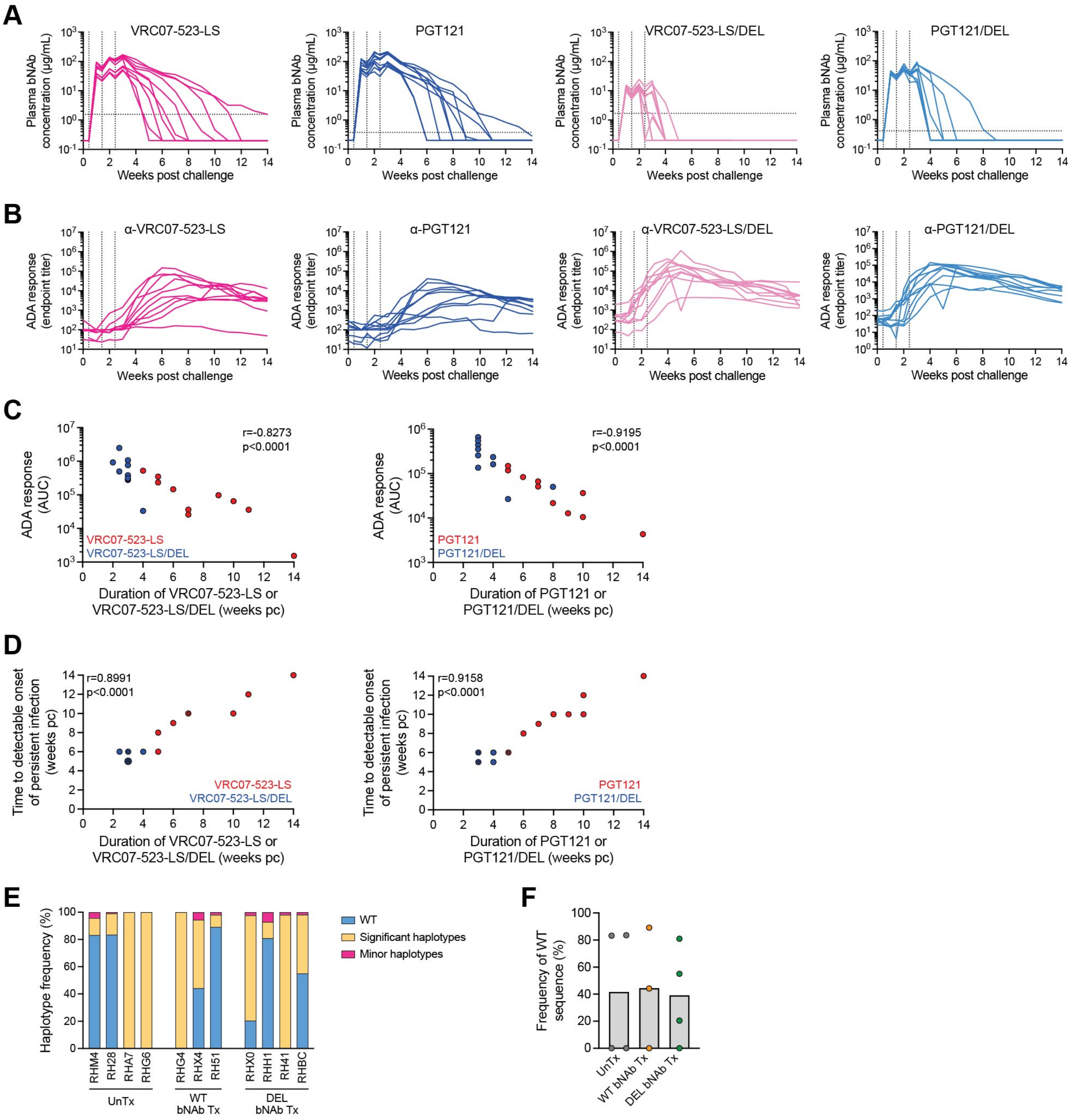

Supplementary Figure 2. bNAb PK, ADA, and SHIV$_{AD8\text{-}EO}$ Env sequencing. (**A, B**) Individual concentrations (A) and ADA responses (B) to VRC07-523-LS (dark pink), PGT121 (dark blue), VRC07-523-LS/DEL (light pink), and PGT121/DEL (light blue), as measured by ELISA in the plasma of monkeys that received either the WT or DEL bNAbs at days 3, 10, and 17 post-SHIV$_{AD8\text{-}EO}$ challenge. Each colored line includes data from one monkey. Horizontal dotted lines indicate the *in vitro* neutralization IC_{80} against SHIV$_{AD8\text{-}EO}$ for each bNAb (A) and vertical dotted lines indicate the timings of bNAb infusions (A, B). (**C**) Correlations between the magnitude of the ADA response against either VRC07-523-LS and VRC07-523-LS/DEL (left) or PGT121 and PGT121/DEL (right) and the duration of the corresponding bNAbs in plasma. (**D**) Correlations between the time to first detectable plasma virus leading to persistent infection and the duration in

plasma of either VRC07-523-LS and VRC07-523-LS/DEL (left) or PGT121 and PGT121/DEL (right). bNAb duration was defined as the last timepoint the bNAb was detected in plasma (or the last timepoint tested, week 14 post-challenge, for the monkey with detectable bNAbs at least until then). (**E, F**) Frequency of the WT Env sequence (E, F) and of significant and minor Env haplotypes (E) in $SHIV_{AD8\text{-}EO}$ isolated from plasma of untreated and bNAb-treated monkeys at the timepoint of peak viremia. Significant and minor haplotypes in a specific sample were defined as haplotypes containing mutations that occurred in that sample with a frequency ≥ 5% or < 5%, respectively. N = 4, 3, and 4 untreated, WT bNAb-treated, and DEL bNAb-treated monkeys (F). Bar graphs show individual data for each monkey (E) and the mean and individual datapoints for each monkey group (F). The Spearman's correlation test was used for correlation analyses (C, D), and the Kruskal-Wallis test was used to detect significant differences between monkey groups (F). N indicates the number of biological replicates (monkeys). Source data are provided in the Source Data file. ADA, anti-drug antibody; AUC, area under the curve; pc, post-challenge; Tx, treatment; UnTx, untreated.

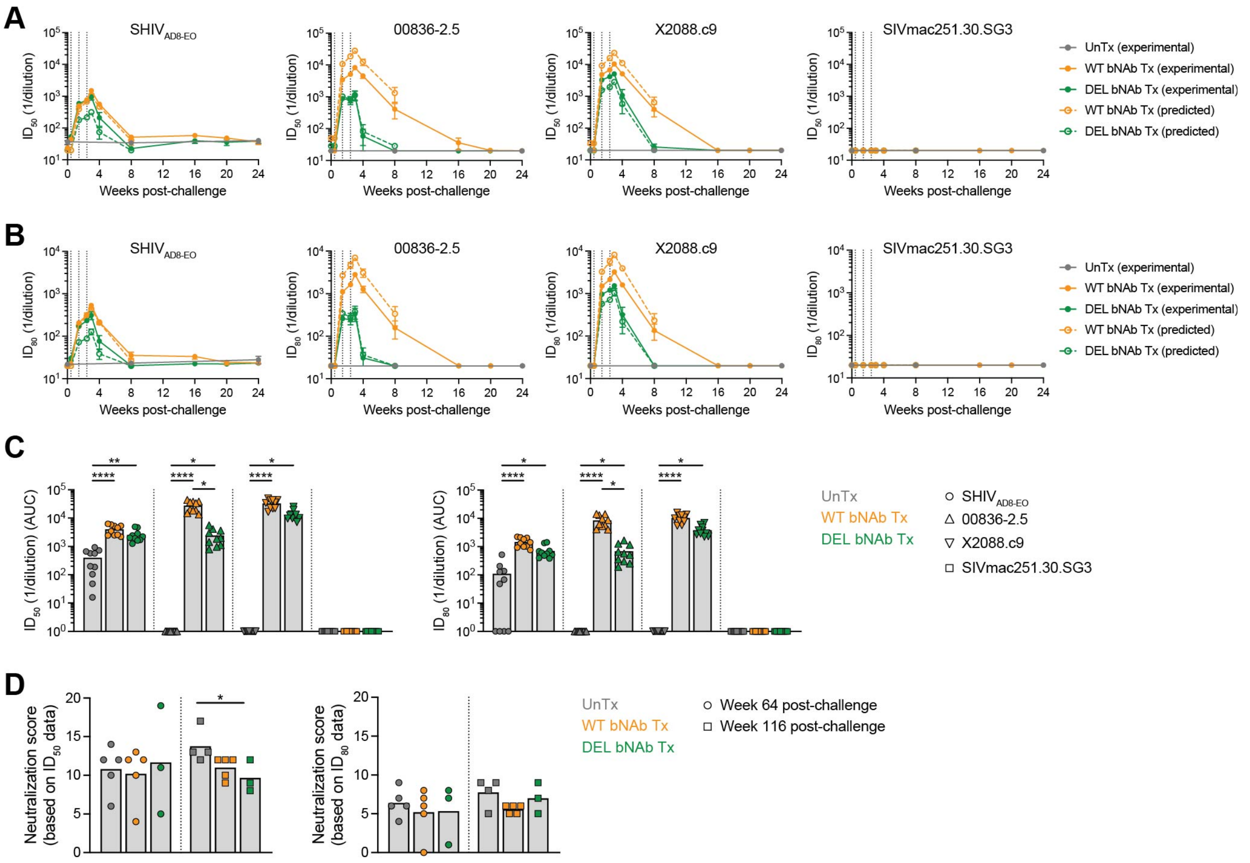

Supplementary Figure 3. Neutralizing activity of plasma from SHIV$_{AD8-EO}$-challenged monkeys on or off early bNAb therapy. (**A, B**) Experimental (solid lines) and predicted (dashed lines) neutralizing titers against the SHIV$_{AD8-EO}$ challenge stock (left), 00836-2.5 (middle left), X2088.c9 (middle right), and SIVmac251.30.SG3 (right), of plasma obtained from SHIV$_{AD8-EO}$-challenged monkeys that were either left untreated or were treated at days 3, 10, and 17 post-challenge with either VRC07-523-LS and PGT121 or VRC07-523-LS/DEL and PGT121/DEL. Plasma neutralizing titers were reported as ID$_{50}$ (A) and ID$_{80}$ (B) values. Predicted titers lower than 20 were plotted as 20 to match the minimum threshold of the experimental data. Vertical dotted lines indicate the timings of bNAb infusions. UnTx (experimental) group, all viruses: n = 10 at all 3 timepoints (week 0, 8, and 24); WT (experimental) group, viruses SHIV$_{AD8-EO}$, 00836-2.5, and X2088.c9: n = 10 except for weeks 16 and 20: n = 7; WT (experimental) group, virus SIVmac251.30.SG3: n = 10 except for days 3, 10 and 17: n = 9 and weeks 16 and 20: n = 7; DEL (experimental) group, all viruses: n = 10 except for weeks 16 and 20: n = 4; WT (predicted) group, all viruses: n = 10 except for week 0: n = 4, and DEL (predicted) group, all viruses: n = 10. (**C**) Plasma neutralizing activity against SHIV$_{AD8-EO}$ (circles), 00836-2.5 (triangles), X2088.c9 (inverted triangles), and SIVmac251.30.SG3 (squares) up to 24 weeks post-challenge, as determined by AUC analysis of ID$_{50}$ (left) and ID$_{80}$ (right) data. Null AUC values (meaning absence of plasma neutralizing activity) were converted to 1 to be visible in graphs with a log$_{10}$ scale. However, statistical analyses were run on the original, unadjusted datasets. N = 10 for each

monkey group and virus. Both ID_{50} and ID_{80} AUC: $P < 0.0001$ for untreated vs. WT bNAb-treated group for viruses $SHIV_{AD8-EO}$, 00836-2.5, and X2088.c9. ID_{50} AUC: p = 0.0082, 0.0290, and 0.0197 for untreated vs. DEL bNAb-treated group for viruses $SHIV_{AD8-EO}$, 00836-2.5, and X2088.c9, respectively, and p = 0.0290 for WT vs. DEL bNAb-treated group for virus 00836-2.5. ID_{80} AUC: p = 0.0264, 0.0290, and 0.0213 for untreated vs. DEL bNAb-treated group for viruses $SHIV_{AD8-EO}$, 00836-2.5, and X2088.c9, respectively, and p = 0.0290 for WT vs. DEL bNAb-treated group for virus 00836-2.5. (**D**) Neutralization score against a wide array of viruses of plasma obtained from untreated and bNAb-treated monkeys at weeks 64 (circles) and 116 (squares) post-challenge. Scores were calculated based on ID_{50} (left) and ID_{80} (right) data. Week 64: n = 5, 5, and 3 untreated, WT bNAb-treated, and DEL bNAb-treated monkeys, respectively; week 116: n = 4, 5, and 3 untreated, WT bNAb-treated, and DEL bNAb-treated monkeys, respectively. P = 0.0421 for untreated vs. DEL bNAb-treated group at week 116 (left). Graphs show the mean ± SEM (A, B), and the mean and individual datapoints (C, D). The Kruskal-Wallis test followed by Dunn's multiple comparison test was used to detect significant differences between monkey groups for each assayed virus (C) and at each timepoint (D). The Mann-Whitney test was used to detect significant differences between timepoints for each monkey group (D). N indicates the number of biological replicates (monkeys). Source data are provided in the Source Data file. AUC, area under the curve; Tx, treatment; UnTx, untreated.

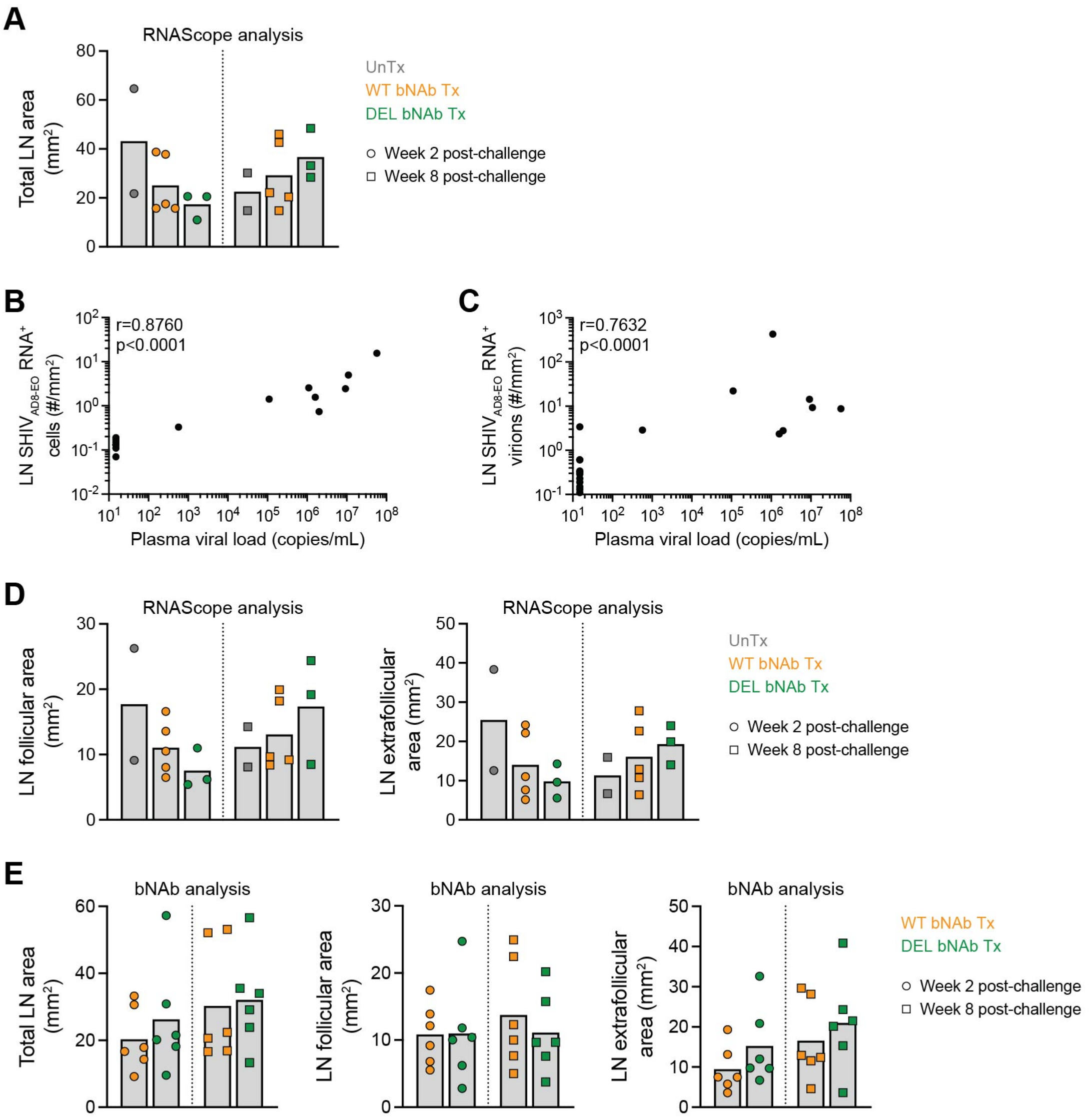

Supplementary Figure 4. Additional analyses of RNAscope and bNAb confocal microscopy data. (**A**) Size of whole LN sections obtained at weeks 2 (circles) and 8 (squares) post-challenge and used for RNAscope analyses. LN sections are from $SHIV_{AD8\text{-}EO}$-challenged monkeys that were either left untreated or were treated at days 3, 10, and 17 post-challenge with either VRC07-523-LS and PGT121 or VRC07-523-LS/DEL and PGT121/DEL. N = 2, 5, and 3 untreated, WT bNAb-treated, and DEL bNAb-treated monkeys, respectively. (**B, C**) Correlations between plasma viral load and either the number of $SHIV_{AD8\text{-}EO}$ RNA^+ cells per mm^2 of LN section (B) or the number of $SHIV_{AD8\text{-}EO}$ RNA^+ virions per mm^2 of LN section (C). (**D**) Size of the follicular (left) and extrafollicular (right) areas of the LN sections obtained at weeks 2 (circles) and 8 (squares) post-challenge and used for RNAscope analyses. N = 2, 5, and 3 untreated, WT bNAb-treated, and DEL bNAb-treated monkeys, respectively. (**E**) Size of whole LN sections (left) as well as of the follicular (middle) and extrafollicular (right) areas of the same LN sections obtained at weeks 2 (circles) and 8 (squares) post-challenge and used for confocal microscopy analyses of infused

bNAbs. N = 6 WT bNAb-treated and 6 DEL bNAb-treated monkeys. Bar graphs show the mean and individual datapoints (A, D, and E). The Mann-Whitney test was used to detect significant differences in the size of LN areas between WT and DEL bNAb-treated monkeys at each timepoint or between timepoints for each bNAb-treated group (A, D, and E). The Spearman's rank correlation test was used for correlation analyses (B, C). N indicates the number of biological replicates (monkeys). Source data are provided in the Source Data file. Tx, treatment; UnTx, untreated.

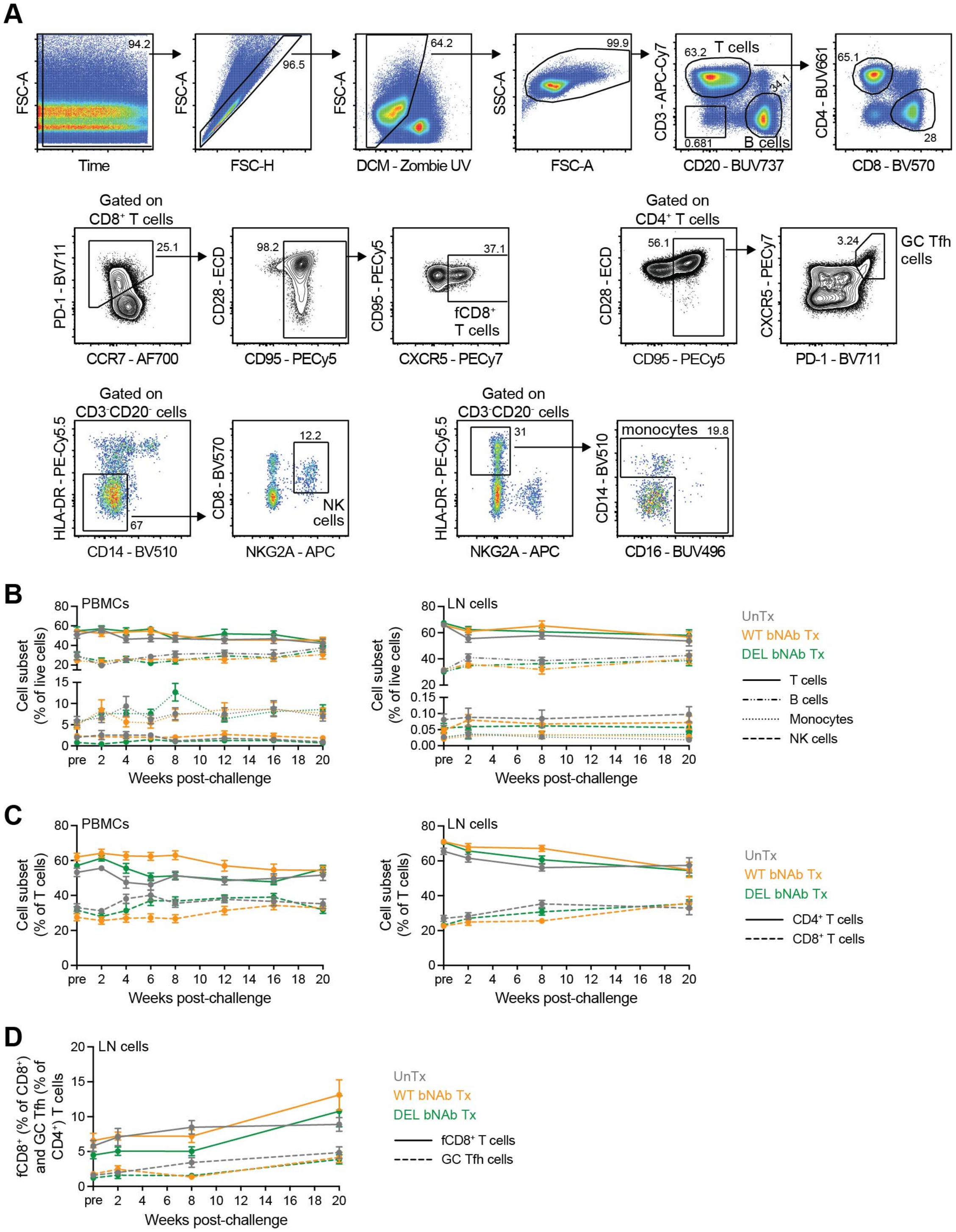

Supplementary Figure 5. Immune cell dynamics in peripheral blood and LNs from $SHIV_{AD8-EO}$-challenged monkeys on or off early bNAb therapy. (**A**) Gating strategy to identify by flow cytometry total T cells, B cells, monocytes, NK cells, $CD4^+$ and $CD8^+$ T cells, $fCD8^+$ T cells, and GC Tfh cells, in monkey LN cells. (**B, C**) Frequency of T cells (solid lines), B cells (dash dotted lines), monocytes (dotted lines), and NK cells (dashed lines) (B), and of $CD4^+$ (solid lines) and

$CD8^+$ (dashed lines) T cells (C) in PBMCs (left) and LN cells (right) from $SHIV_{AD8-EO}$-challenged monkeys that were either left untreated or were treated at days 3, 10, and 17 post-challenge with either VRC07-523-LS and PGT121 or VRC07-523-LS/DEL and PGT121/DEL. PBMCs, each timepoint: n = 9, 8, and 7 untreated, WT bNAb-treated, and DEL bNAb-treated monkeys, respectively. LN cells: n = 8, 6, 9, and 4 untreated monkeys at weeks 0, 2, 8, and 20, respectively; n = 8 and 7 WT and DEL bNAb-treated monkeys, respectively, at each timepoint. (**D**) Frequency of $fCD8^+$ T cells (solid lines) and GC Tfh cells (dashed lines) in LN cells from untreated and bNAb-treated monkeys. N = 8, 6, 9, and 4 untreated monkeys at weeks 0, 2, 8, and 20, respectively; n = 8 and 7 WT and DEL bNAb-treated monkeys, respectively, at each timepoint. Graphs show the mean ± SEM (B-D). N indicates the number of biological replicates (monkeys). Source data are provided in the Source Data file. DCM, dead cell marker; $fCD8^+$, follicular $CD8^+$; GC, germinal center; LN, lymph node; PBMCs, peripheral blood mononuclear cells; Tfh, T follicular helper; Tx, treatment; UnTx, untreated.

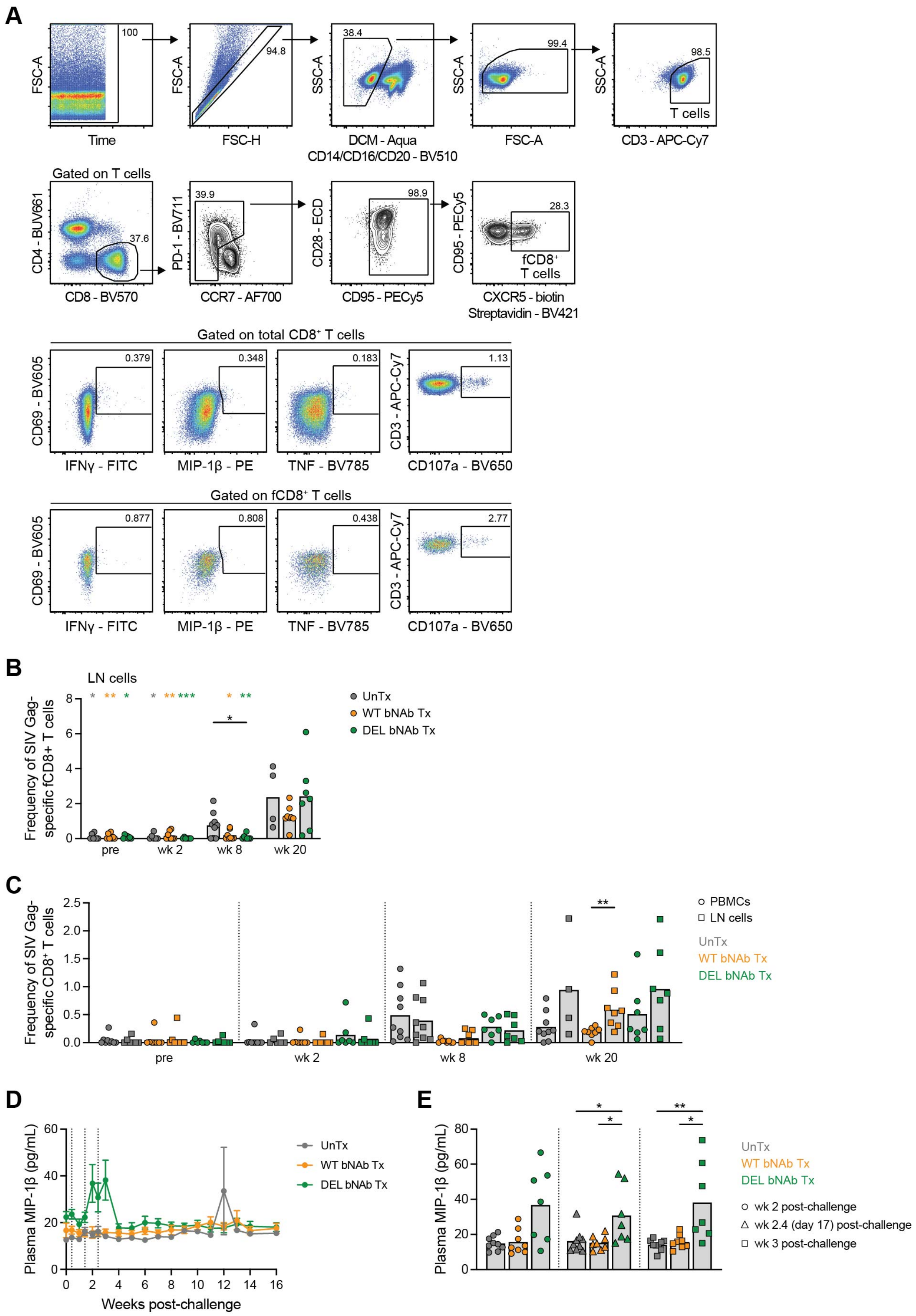

Supplementary Figure 6. SIV Gag-specific CD8$^+$ T cell responses and plasma levels of MIP-1β in SHIV$_{AD8\text{-}EO}$-challenged monkeys on or off early bNAb therapy. (A) Gating strategy to

identify by flow cytometry total $CD8^+$ and $fCD8^+$ T cells in monkey LN cells, and representative example of the co-expression of CD69 and IFNγ, MIP-1β, or TNF, and of CD107a expression, by LN total $CD8^+$ and $fCD8^+$ T cells after *in vitro* stimulation with SIV Gag peptide pool. (**B**) Frequency of SIV Gag-specific $fCD8^+$ T cells in LN cells from $SHIV_{AD8-EO}$-challenged monkeys that were either left untreated or were treated at days 3, 10, and 17 post-challenge with either VRC07-523-LS and PGT121 or VRC07-523-LS/DEL and PGT121/DEL. SIV Gag-specific $fCD8^+$ T cells were defined by flow cytometry as $fCD8^+$ T cells either co-expressing CD69 and IFNγ, MIP-1β, or TNF, or expressing CD107a, after *in vitro* stimulation of LN cells with SIV Gag peptide pool. N = 7, 6, 9, and 4 untreated monkeys at pre, week 2, 8, and 20, respectively; n = 8 and 7 WT and DEL bNAb-treated monkeys, respectively, at each timepoint. P = 0.0396 for untreated vs. DEL bNAb-treated group at week 8. (**C**) Frequency of SIV Gag-specific $CD8^+$ T cells in PBMCs (circles) and LN cells (squares) from untreated and bNAb-treated monkeys after *in vitro* stimulation with SIV Gag peptide pool. PBMCs: n = 9, 8, and 7 untreated, WT bNAb-treated, and DEL bNAb-treated monkeys, respectively. LN cells: n = 7, 6, 9, and 4 untreated monkeys at pre, week 2, 8, and 20, respectively; n = 8 and 7 WT and DEL bNAb-treated monkeys, respectively, at each timepoint. P = 0.0031 for PBMCs vs. LN cells at week 20 in WT bNAb-treated group; two-sided p-value. (**D, E**) Plasma levels of MIP-1β longitudinally throughout the first 16 weeks post-challenge (D) and at weeks 2 (circles), 2.4 (day 17, triangles), and 3 (squares) post-challenge (E) in untreated and bNAb-treated monkeys, as measured by Luminex. Vertical dotted lines indicate the timings of bNAb infusions (D). N = 9 untreated monkeys except for weeks 9, 10, 11, 13, and 14: n = 4; n = 8 and 7 WT and DEL bNAb-treated monkeys, respectively (D). N = 9, 8, and 7 untreated, WT bNAb-treated, and DEL bNAb-treated monkeys, respectively. P = 0.0325 and p = 0.0074 for untreated vs. DEL bNAb-treated group at weeks 2.4 and 3, respectively; P = 0.0465 and p = 0.0395 for WT vs. DEL bNAb-treated group at weeks 2.4 and 3, respectively. (E) Graphs show the mean and individual datapoints (B, C, and E) and the mean ± SEM (D). The Kruskal-Wallis test followed by Dunn's multiple comparison test was used to detect significant differences between monkey groups at each timepoint (B, E) and between timepoints for each monkey group (colored statistical results in B). Statistical results indicated in gray, orange, and green, denote differences from week 20 post-challenge for all monkey groups (B). The Mann-Whitney test was used to detect significant differences between PBMCs and LN cells for each monkey group and at each timepoint (C). N indicates the number of biological replicates (monkeys). Source data are provided in the Source Data file. DCM, dead cell marker; $fCD8^+$, follicular $CD8^+$; LN, lymph node; PBMCs, peripheral blood mononuclear cells; pre, pre-challenge; Tx, treatment; UnTx, untreated; wk, week.

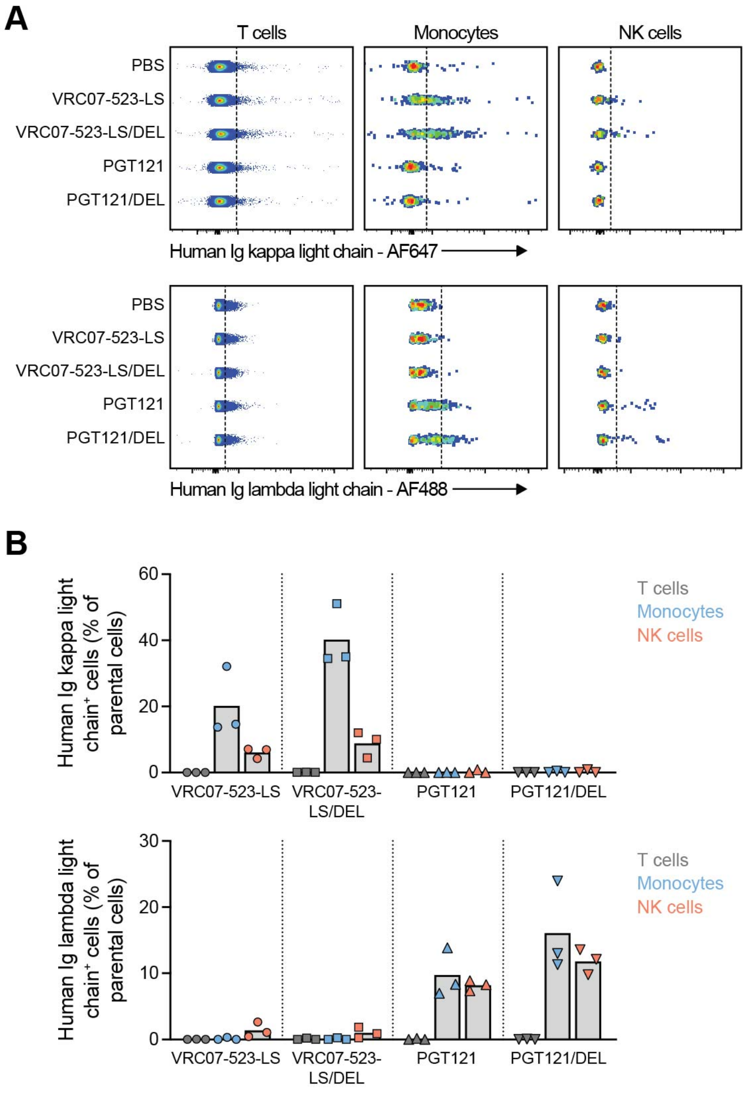

Supplementary Figure 7. *In vitro* coating of monkey LN cells with anti-HIV-1 bNAbs. (**A**) Representative example of the flow cytometry staining to detect human Ig kappa (top) and human Ig lambda (bottom) light chain in T cells (left), monocytes (middle), and NK cells (right), following incubation of monkey LN cells with VRC07-523-LS, VRC07-523-LS/DEL, PGT121, PGT121/DEL, or PBS as negative control. Vertical dotted lines indicate gating for human Ig kappa light chain$^+$ and human Ig lambda light chain$^+$ events. (**B**) Frequency of human Ig kappa light chain$^+$ (top) and human Ig lambda light chain$^+$ (bottom) T cells, monocytes, and NK cells, following incubation of monkey LN cells with VRC07-523-LS (circles), VRC07-523-LS/DEL (squares), PGT121 (triangles), or PGT121/DEL (inverted triangles). For each cell population, the frequency of positive events upon incubation of LN cells with each bNAb was background subtracted using the residual frequency of positive events upon incubation with PBS (negative control). N = 3 monkeys for each cell type and bNAb. Bar graphs show the mean and individual datapoints (B). N indicates the number of biological replicates (monkeys). Source data are provided in the Source Data file. Ig, immunoglobulin.

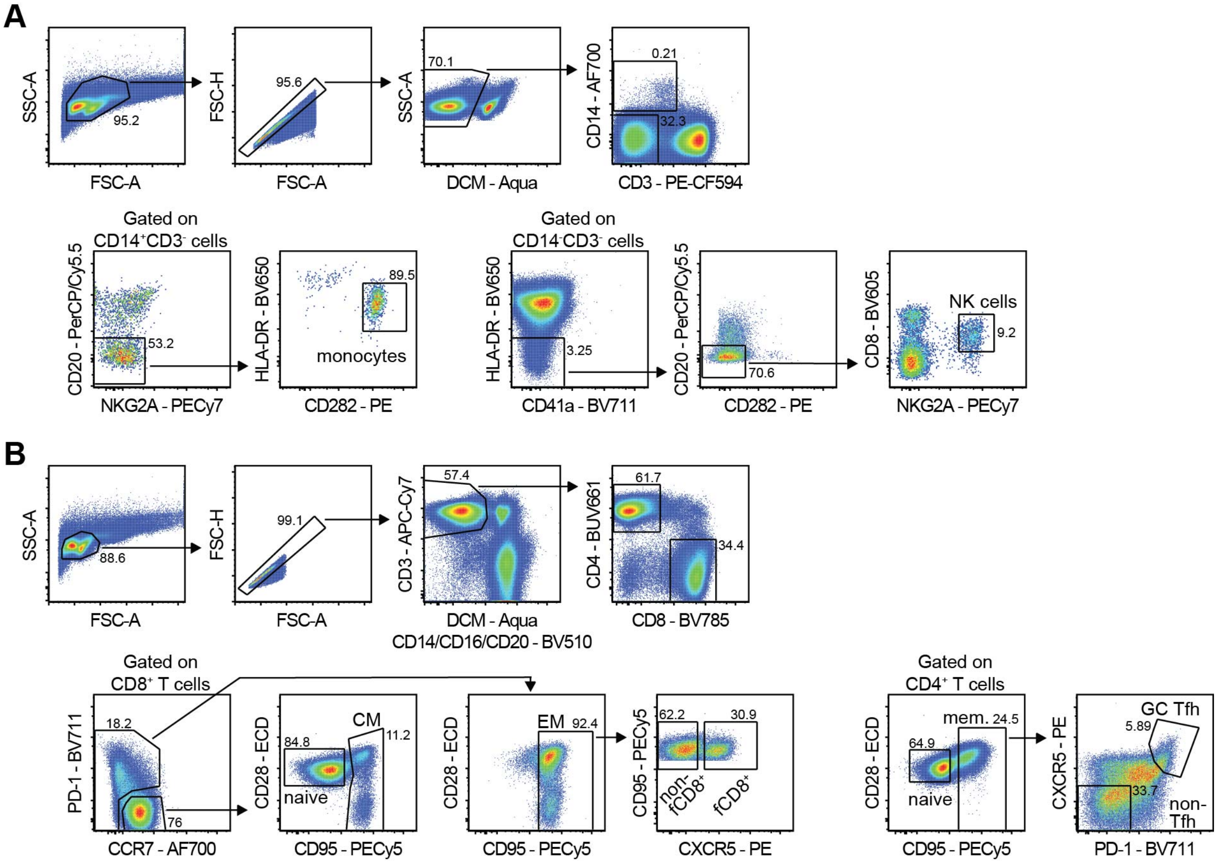

Supplementary Figure 8. Gating strategies for sorting of LN cells for RNA sequencing. (**A, B**) Gating strategies to identify and sort monocytes and NK cells (A), total CD8$^+$ T cells and subsets (naïve, central memory, effector memory, fCD8$^+$, and non-fCD8$^+$ cells), and total CD4$^+$ T cells and subsets (naïve, memory, GC Tfh, and non-Tfh cells) (B). Cell subsets were sorted from LN cells of $SHIV_{AD8\text{-}EO}$-challenged monkeys that were either left untreated or were treated at days 3, 10, and 17 post-challenge with either VRC07-523-LS and PGT121 or VRC07-523-LS/DEL and PGT121/DEL. CM, central memory; DCM, dead cell marker; EM, effector memory; fCD8$^+$, follicular CD8$^+$; GC, germinal center; mem., memory; Tfh, T follicular helper.

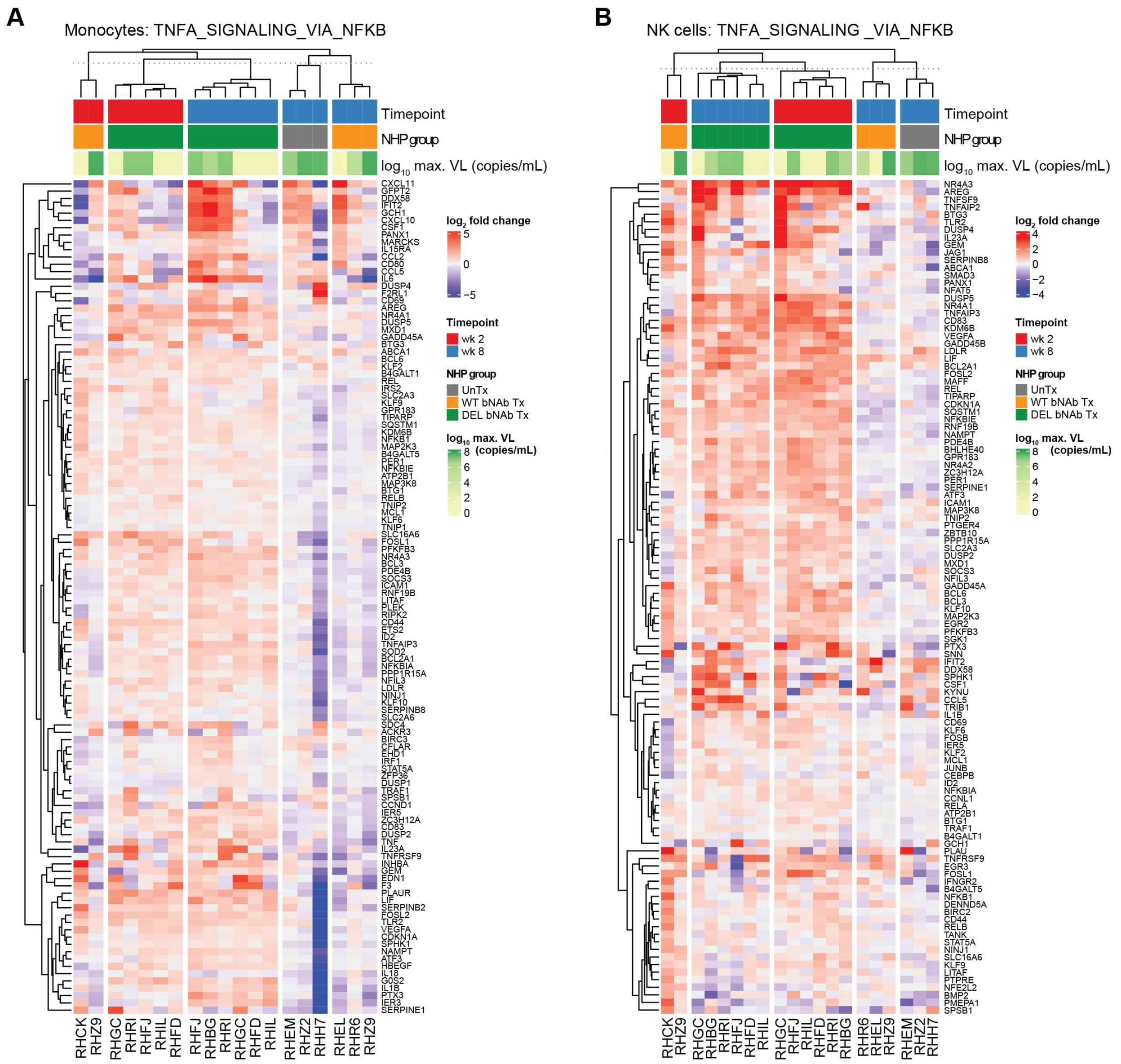

Supplementary Figure 9. TNF signaling via NF-κB in LN monocytes and NK cells from SHIV$_{AD8\text{-}EO}$-challenged monkeys on or off early bNAb therapy. (**A, B**) Heatmaps showing the relative expression of genes involved in TNF signaling via NF-κB (from the Hallmark geneset HALLMARK_TNFA_SIGNALING_VIA_NFKB) as determined by transcriptomics of sorted LN monocytes (A) and NK cells (B) from SHIV$_{AD8\text{-}EO}$-challenged monkeys that were either left untreated or were treated at days 3, 10, and 17 post-challenge with either VRC07-523-LS and PGT121 or VRC07-523-LS/DEL and PGT121/DEL. The genes most strongly contributing to the enrichment signal of this pathway (i.e., leading edges) are presented in rows and the samples profiled are shown in columns. Cells correspond to the $\log_2$ fold-change in gene expression at each depicted timepoint post-challenge when compared to the pre-challenge timepoint, with a red-white-blue gradient indicating induction, lack of differential expression, and repression of gene expression, respectively. The timepoints, NHP groups, and $\log_{10}$ plasma viral loads are included

as column annotations in the heatmaps. Source data are provided in the Source Data file. Max., maximum; NHP, non-human primate; Tx, treatment; UnTx, untreated; VL, viral load; wk, week.

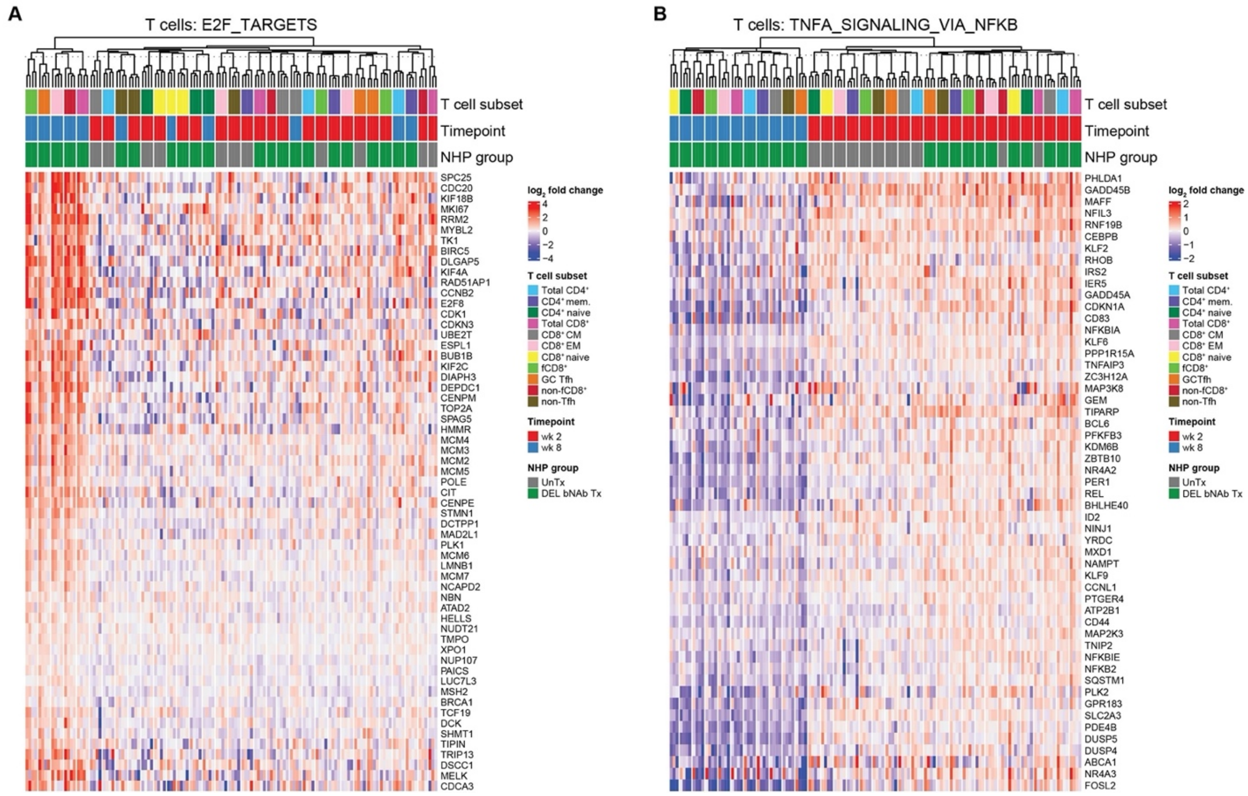

Supplementary Figure 10. E2F and TNF signaling via NF-κB in LN T cell subsets from SHIV$_{\text{AD8-EO}}$-challenged monkeys on or off early bNAb therapy. (**A, B**) Heatmaps showing the relative expression of genes involved in E2F signaling (from the Hallmark geneset HALLMARK_E2F_TARGETS) (A) and TNF signaling via NF-κB (from the Hallmark geneset HALLMARK_TNFA_SIGNALING_VIA_NFKB) (B) as determined by transcriptomics of sorted LN T cell subsets from SHIV$_{\text{AD8-EO}}$-challenged monkeys that were either left untreated or were treated at days 3, 10, and 17 post-challenge with either VRC07-523-LS and PGT121 or VRC07-523-LS/DEL and PGT121/DEL. The genes most strongly contributing to the enrichment signal of each of these pathways (i.e., leading edges) are presented in rows and the samples profiled are shown in columns. Cells correspond to the $\log_2$ fold-change in gene expression at each depicted timepoint post-challenge when compared to the pre-challenge timepoint, with a red-white-blue gradient indicating induction, lack of differential expression, and repression of gene expression, respectively. The T cell subsets, timepoints, and NHP groups are included as column annotations in the heatmaps. Source data are provided in the Source Data file. CM, central memory; EM, effector memory; fCD8$^+$, follicular CD8$^+$; GC, germinal center; mem., memory; NHP, non-human primate; Tfh, T follicular helper; Tx, treatment; UnTx, untreated; wk, week.

SUPPLEMENTARY TABLES

Supplementary Table 1. Plasma viral load in copies/mL in SHIV$_{AD8\text{-}EO}$-challenged uninfected monkeys, as determined by ultrasensitive SIV Gag RNA qRT-PCR

<table>
<tr><th rowspan="2">Time post-challenge (weeks)</th><th>UnTx</th><th colspan="2">WT bNAb Tx</th><th colspan="3">DEL bNAb Tx</th></tr>
<tr><th>RH81</th><th>RHCK</th><th>RHL9</th><th>RHFD</th><th>RHGC</th><th>RHIL</th></tr>
<tr><td>pre-challenge</td><td rowspan="2">4</td><td rowspan="2">Below 1</td><td rowspan="2">Below 1</td><td rowspan="2">Below 1</td><td rowspan="2">Below 1</td><td rowspan="2">Below 1</td></tr>
<tr><td>0</td></tr>
<tr><td>0.43</td><td rowspan="5">Below 1</td><td rowspan="5">Below 1</td><td rowspan="5">Below 1</td><td rowspan="5">Below 1</td><td rowspan="5">Below 1</td><td rowspan="5">Below 1</td></tr>
<tr><td>1</td></tr>
<tr><td>1.43</td></tr>
<tr><td>2</td></tr>
<tr><td>2.43</td></tr>
<tr><td>3</td><td rowspan="4">Below 1</td><td rowspan="4">Below 1</td><td rowspan="4">Below 1</td><td rowspan="4">Below 1</td><td rowspan="4">Below 1</td><td rowspan="4">Below 1</td></tr>
<tr><td>4</td></tr>
<tr><td>5</td></tr>
<tr><td>6</td></tr>
<tr><td>7</td><td rowspan="2">Below 1*</td><td rowspan="4">Below 1</td><td rowspan="4">Below 1</td><td rowspan="4">Below 1</td><td rowspan="4">Below 1</td><td rowspan="4">Below 1</td></tr>
<tr><td>8</td></tr>
<tr><td>9</td><td>N/A</td></tr>
<tr><td>10</td><td>N/A</td></tr>
<tr><td>11</td><td>N/A</td><td rowspan="4">Below 1</td><td rowspan="4">Below 1</td><td rowspan="4">Below 1</td><td rowspan="4">Below 1</td><td rowspan="4">Below 1</td></tr>
<tr><td>12</td><td>Below 1*</td></tr>
<tr><td>13</td><td>N/A</td></tr>
<tr><td>14</td><td>N/A</td></tr>
<tr><td>16</td><td rowspan="2">Below 1*</td><td>N.D.</td><td>N.D.</td><td>N.D.</td><td>N.D.</td><td>N.D.</td></tr>
<tr><td>20</td><td>Below 1</td><td>Below 1</td><td>Below 1</td><td>Below 1</td><td>Below 1</td></tr>
<tr><td>24</td><td>Below 4†</td><td>Below 1</td><td>Below 1</td><td>Below 1</td><td>Below 1</td><td>Below 1</td></tr>
<tr><td>32</td><td>Below 1</td><td>Below 1</td><td>Below 1</td><td>Below 1</td><td>Below 1</td><td>Below 1</td></tr>
</table>

*To reach the desired volume of plasma for ultrasensitive qRT-PCR, samples from multiple, close timepoints were pooled before the assay. Samples from animal RH81 at weeks 7, 8, 12, 16, and 20 post-challenge were all pooled before the assay and the reported value (below 1) came from only one assayed pool.

†The detection limit of the assay was 1 copy/mL, with the exception of sample from animal RH81, week 24 post-challenge, where the detection limit was 4 copies/mL.

N/A, not applicable (samples were not collected at these timepoints); N.D., not determined; Tx, treatment; UnTx, untreated.

Supplementary Table 2. Plasma viral load in copies/mL after anti-CD8β mAb infusion in $SHIV_{AD8-EO}$-challenged uninfected monkeys, as determined by ultrasensitive SIV Gag RNA qRT-PCR

<table>
<tr><th rowspan="2">Time post-anti-CD8β mAb infusion (days)</th><th>UnTx</th><th>WT bNAb Tx</th><th colspan="3">DEL bNAb Tx</th></tr>
<tr><th>RH81</th><th>RHCK</th><th>RHFD[¶]</th><th>RHGC[¶]</th><th>RHIL</th></tr>
<tr><td>-14* or -10[†]</td><td rowspan="3">Below 1</td><td rowspan="3">Below 1</td><td>Below 3</td><td>31</td><td rowspan="3">Below 1</td></tr>
<tr><td>-7* or -5[†]</td><td>Below 3</td><td>Below 3</td></tr>
<tr><td>0, 0h</td><td>Below 2</td><td>Below 2</td></tr>
<tr><td>0, 1h</td><td rowspan="2">Below 1</td><td rowspan="2">Below 1</td><td>10</td><td>Below 2</td><td rowspan="2">Below 1</td></tr>
<tr><td>0, 6h</td><td>Below 2</td><td>Below 2</td></tr>
<tr><td>1</td><td rowspan="2">Below 1</td><td rowspan="2">5</td><td>N/A</td><td>N/A</td><td>N/A</td></tr>
<tr><td>2</td><td>Below 2</td><td rowspan="2">Below 1</td><td rowspan="2">Below 1</td></tr>
<tr><td>4</td><td>N/A</td><td>N/A</td><td>Below 2</td></tr>
<tr><td>7</td><td rowspan="2">Below 1[‡]</td><td rowspan="2">Below 1[‡]</td><td>Below 2</td><td rowspan="2">Below 1</td><td rowspan="2">4</td></tr>
<tr><td>9</td><td>Below 2</td></tr>
<tr><td>11</td><td>N/A</td><td>N/A</td><td>Below 2</td><td rowspan="2">Below 1</td><td rowspan="2">Below 1</td></tr>
<tr><td>14</td><td>Below 1[‡]</td><td>Below 1[‡]</td><td>Below 2</td></tr>
<tr><td>17</td><td>N/A</td><td>N/A</td><td>Below 2</td><td rowspan="2">Below 1</td><td rowspan="2">Below 1</td></tr>
<tr><td>21</td><td>Below 1[‡]</td><td>Below 1[‡]</td><td>Below 2</td></tr>
</table>

[*]Timepoints for animals RH81 and RHCK; [†]Timepoints for animals RHFD, RHGC, and RHIL.

[‡]To reach the desired volume of plasma for ultrasensitive qRT-PCR, samples from multiple, close timepoints were pooled before the assay. Samples from animals RH81 and RHCK at days 7, 9, 14, and 21 post-anti-CD8β mAb infusion were pooled before the assay and the reported values (below 1) came from only one assayed pool per animal.

[¶]The detection limit of the assay was 1 copy/mL, with the exception of samples from animals RHFD and RHGC, where the detection limit was 1, 2, or 3 copies/mL.

N/A, not applicable (samples were not collected at these timepoints); Tx, treatment; UnTx, untreated.

Supplementary Table 3. Levels of SIV Gag DNA and RNA after anti-CD8β mAb infusion in PBMCs from $SHIV_{AD8-EO}$-challenged and DEL bNAb-treated uninfected monkeys

Time post-anti-CD8β mAb infusion (days)	RHFD		RHGC		RHIL	
	SIV Gag DNA (copies/10^6 cell eq.)	SIV Gag RNA (copies/10^6 cell eq.)	SIV Gag DNA (copies/10^6 cell eq.)	SIV Gag RNA (copies/10^6 cell eq.)	SIV Gag DNA (copies/10^6 cell eq.)	SIV Gag RNA (copies/10^6 cell eq.)
**-5**	2.2	2.2	1.7	1.7	2.0	2.0
**0, 0h**	2.6	2.6	2.4	2.4	2.2	2.2
**0, 1h**	3.6	3.6	3.6	3.6	3.6	3.6
**0, 6h**	3.5	3.5	3.2	2.9	3.8	3.8
**2**	5.4	5.4	2.5	2.5	2.8	2.8
**4**	3.6	3.6	2.0	2.0	1.6	1.6
**7**	2.8	2.8	3.2	3.2	2.3	2.3
**9**	2.5	3.2	2.8	2.8	2.9	2.9
**11**	2.7	2.8	2.8	2.9	2.0	2.0
**14**	2.6	2.6	2.3	2.3	3.0	3.0
**17**	2.9	2.9	2.3	2.3	2.5	2.5
**21**	3.3	3.3*	3.0	3.0	2.7	2.7

*Only result above the assay threshold.
eq., equivalents.

Supplementary Table 4. Plasma samples used for $SHIV_{AD8-EO}$ sequencing and description of major Env haplotypes and significant minor mutations in each sample

Group	Animal ID	Time of peak viremia (weeks post-challenge)	Peak plasma viral load (copies/mL)	SGSs recovered (#)	Major haplotype		Significant minor mutations*	
					Haplotype	Frequency (%)	Mutation	Frequency (%)
UnTx	**RHM4**	2.4 (day 17)	6.5E+07	2329	WT	83.2	S662G<br>M512V	7.0<br>6.1
	**RH28**	2.4 (day 17)	3.3E+07	1647	WT	83.5	S662G<br>M512V	8.5<br>7.6
	**RHA7**	6	1.1E+05	286	L121I + N810S	86.0	M512V<br>S662G	7.7<br>7.3
	**RHG6**	21	7.8E+05	659	T62P + G731D + D753N + R755W	35.5	S55T<br>D56N	17.3<br>10.4
WT bNAb Tx	**RHG4**	12	1.9E+07	1079	K225R	67.8	S662G<br>M512V	15.2<br>14.5
	**RHX4**	8	1.3E+06	882	WT	44.2	G744D<br>M512V<br>L749F<br>S662G	26.9<br>13.6<br>6.9<br>6.2
	**RH51**	11	2.3E+07	1498	WT	89.1	M512V	5.4
DEL bNAb Tx	**RHX0**	8	4.8E+06	977	S662G	68.4	M512V	8.3
	**RHH1**	6	3.8E+07	1358	WT	80.9	M512V<br>S662G	7.4<br>5.0
	**RH41**	7	7.3E+06	1152	Q795R	80.4	M512V<br>S662G	9.0<br>6.7
	**RHBC**	8	4.9E+06	888	WT	55.1	G731D<br>M512V<br>I190M<br>S662G	32.1<br>8.1<br>6.2<br>5.1

*Only mutations that occured with a frequency ≥ 5% in that specific sample are shown.
SGSs, single-genome sequences; Tx, treatment; UnTx, untreated.

Supplementary Table 5. Neutralization potency of anti-HIV-1 bNAbs against viruses used in neutralization assays (results are shown in Supplementary Figure 3, A-C)

bNAb	IC_{50} (µg/mL)				IC_{80} (µg/mL)			
	$SHIV_{AD8-EO}$	00836-2.5	X2088.c9	SIVmac251.30.SG3	$SHIV_{AD8-EO}$	00836-2.5	X2088.c9	SIVmac251.30.SG3
**VRC07-523-LS**	0.723	0.004	>50	>50	1.57	0.016	>50	>50
**VRC07-523-LS/DEL**	0.731	0.007	36.3	>50	1.68	0.021	>50	>50
**PGT121**	0.167	>50	0.006	>50	0.375	>50	0.017	>50
**PGT121/DEL**	0.168	12.2	0.018	>50	0.413	>50	0.049	>50

Values highlighted in red, yellow, and green indicate high, medium, and low potency, respectively.

Supplementary Table 6. Neutralizing activity of plasma from untreated and bNAb-treated monkeys at weeks 64 and 116 post-$SHIV_{AD8-EO}$ challenge. Data are reported as ID_{50}

	Clade	B SHIV		B					C						A	
	Tier	N/A		1B	1B	2	2	2	1A	1B	2	2	2	2	1B	
Group	Animal ID	$SHIV_{DH12}$ clone 7 V3 AD8	$SHIV_{AD8-E0}$	HXB2	6535.3	JR-CSF	JRFL	WITO	MW965.26	ZM109.4	CAP256 SU	DU156.12	ZM233.6	CH505	Q23.17	Neutralization Score
Week 64 post-challenge																
UnTx	RHH7	2249	24	290	138	332	25	<20	2296	<20	<20	<20	<20	<20	<20	12
	RHEM	3244	34	276	59	93	22	<20	1539	<20	<20	<20	<20	<20	<20	10
	RHZ2	6037	23	262	260	282	32	<20	3948	<20	<20	<20	<20	<20	<20	12
	RHAM	1355	<20	35	<20	42	<20	<20	398	<20	<20	<20	<20	<20	<20	6
	RHR8	5458	66	445	43	227	23	<20	2676	<20	<20	118	<20	<20	<20	14
WT bNAb Tx	RHEL	3320	122	83	51	90	<20	<20	520	<20	<20	<20	<20	<20	<20	10
	RHR6	268	23	31	67	<20	<20	<20	44	<20	<20	<20	<20	<20	<20	4
	RHZ9	3429	36	300	139	181	<20	<20	2128	<20	<20	21	<20	<20	<20	12
	RHDL	2089	25	153	226	312	<20	<20	3727	<20	<20	<20	<20	<20	<20	12
	RHIV	2530	46	703	117	419	<20	<20	2280	<20	<20	<20	<20	<20	<20	13
DEL bNAb Tx	RHRI	641	23	44	21	80	<20	<20	47	<20	<20	<20	<20	<20	<20	5
	RHBG	1606	226	497	256	317	55	<20	2047	<20	<20	192	126	<20	<20	19
	RHFJ	2892	28	228	43	172	26	<20	1705	<20	<20	<20	<20	<20	<20	11
Week 116 post-challenge																
UnTx	RHH7	3300	85	385	274	142	21	<20	2559	<20	<20	<20	<20	<20	<20	13
	RHEM	N/A	N/A	N/A	N/A	N/A	N/A	N/A	N/A	N/A	N/A	N/A	N/A	N/A	N/A	N/A
	RHZ2	3018	68	262	216	164	<20	<20	4054	<20	<20	<20	<20	<20	<20	13
	RHAM	1004	102	108	57	73	<20	<20	1106	<20	<20	<20	<20	<20	<20	12
	RHR8	3435	130	923	107	163	79	<20	8850	<20	<20	41	63	<20	<20	17
WT bNAb Tx	RHEL	963	43	175	46	50	<20	<20	1559	<20	<20	<20	<20	<20	<20	10
	RHR6	1170	38	125	49	62	<20	<20	334	<20	<20	27	<20	<20	<20	9
	RHZ9	5668	47	347	67	213	<20	<20	2377	<20	<20	<20	<20	<20	<20	12
	RHDL	2360	38	187	231	171	23	<20	2814	<20	<20	<20	<20	<20	24	12
	RHIV	3381	32	440	146	243	<20	<20	1232	<20	<20	<20	<20	<20	<20	12
DEL bNAb Tx	RHRI	917	55	179	33	55	<20	<20	268	<20	<20	<20	<20	<20	<20	8
	RHBG	1248	120	362	<20	49	<20	<20	972	<20	<20	<20	802	<20	<20	12
	RHFJ	2277	29	745	29	77	24	<20	4001	<20	<20	<20	<20	<20	<20	9

Color scheme for ID_{50} values represents neutralization potency: 40 - 99, green; 100 - 999, yellow; ≥ 1000, red.
N/A, not applicable; Tx, treatment; UnTx, untreated.

Supplementary Table 7. Neutralizing activity of plasma from untreated and bNAb-treated monkeys at weeks 64 and 116 post-$SHIV_{AD8\text{-}EO}$ challenge. Data are reported as ID_{80}

	Clade	B SHIV		B					C						A	
	Tier	N/A		1B	1B	2	2	2	1A	1B	2	2	2	2	1B	
Group	Animal ID	$SHIV_{DH12}$ clone 7 V3 AD8	$SHIV_{AD8\text{-}E0}$	HXB2	6535.3	JR-CSF	JRFL	WITO	MW965.26	ZM109.4	CAP256 SU	DU156.12	ZM233.6	CH505	Q23.17	Neutralization Score
**Week 64 post-challenge**																
	RHH7	235	<20	130	30	99	<20	<20	506	<20	<20	<20	<20	<20	<20	7
	RHEM	284	<20	138	<20	29	<20	<20	491	<20	<20	<20	<20	<20	<20	6
UnTx	RHZ2	596	<20	135	59	85	<20	<20	1984	<20	<20	<20	<20	<20	<20	9
	RHAM	137	<20	23	<20	<20	<20	<20	144	<20	<20	<20	<20	<20	<20	4
	RHR8	396	23	210	<20	39	<20	<20	721	<20	<20	<20	<20	<20	<20	6
	RHEL	214	40	58	<20	27	<20	<20	99	<20	<20	<20	<20	<20	<20	5
	RHR6	23	<20	<20	<20	<20	<20	<20	21	<20	<20	<20	<20	<20	<20	0
WT bNAb Tx	RHZ9	253	<20	136	31	30	<20	<20	535	<20	<20	<20	<20	<20	<20	6
	RHDL	192	<20	100	42	68	<20	<20	845	<20	<20	<20	<20	<20	<20	8
	RHIV	208	23	384	33	73	<20	<20	688	<20	<20	<20	<20	<20	<20	7
	RHRI	75	<20	20	<20	<20	<20	<20	<20	<20	<20	<20	<20	<20	<20	1
DEL bNAb Tx	RHBG	186	88	345	<20	48	<20	<20	804	<20	<20	<20	<20	<20	<20	8
	RHFJ	270	<20	131	<20	59	<20	<20	501	<20	<20	<20	<20	<20	<20	7
**Week 116 post-challenge**																
	RHH7	323	51	167	40	32	<20	<20	365	<20	<20	<20	<20	<20	<20	8
	RHEM	N/A	N/A	N/A	N/A	N/A	N/A	N/A	N/A	N/A	N/A	N/A	N/A	N/A	N/A	N/A
UnTx	RHZ2	255	36	104	47	40	<20	<20	2105	<20	<20	<20	<20	<20	<20	9
	RHAM	135	74	37	<20	<20	<20	<20	455	<20	<20	<20	<20	<20	<20	5
	RHR8	251	50	543	27	58	32	<20	2343	<20	<20	<20	<20	<20	<20	9
	RHEL	65	23	108	<20	<20	<20	<20	197	<20	<20	<20	<20	<20	<20	5
	RHR6	101	20	64	<20	25	<20	<20	100	<20	<20	<20	<20	<20	<20	5
WT bNAb Tx	RHZ9	422	25	155	<20	28	<20	<20	237	<20	<20	<20	<20	<20	<20	6
	RHDL	246	<20	86	35	68	<20	<20	845	<20	<20	<20	<20	<20	<20	6
	RHIV	240	20	199	<20	36	<20	<20	465	<20	<20	<20	<20	<20	<20	6
	RHRI	106	<20	99	<20	<20	<20	<20	103	<20	<20	<20	<20	<20	<20	5
DEL bNAb Tx	RHBG	124	84	206	<20	<20	<20	<20	454	<20	<20	<20	178	<20	<20	9
	RHFJ	273	<20	260	<20	24	<20	<20	1835	<20	<20	<20	<20	<20	<20	7

Color scheme for ID_{80} values represents neutralization potency: 40 - 99, green; 100 - 999, yellow; ≥ 1000, red.
N/A, not applicable; Tx, treatment; UnTx, untreated.

Supplementary Table 8. ***FCGR2*** **and** ***FCGR3*** **genotype of the rhesus macaques used in this study**

Animal ID	*FCGR2*	*FCGR3*
**UnTx**		
RHH7	2A-1/2A-2	3A-1
RHEM	2A-2/2A-4	3A-2
RHZ2	2A-1	3A-1/3A-2
RHAM	2A-1	3A-1/3A-2
RH81*	2A-1/2A-2	3A-2/3A-3
RHR8	2A-1/2A-2	3A-2/3A-3
RHM4	2A-1/2A-2	3A-1
RH28	2A-1	3A-2
RHA7	2A-2	3A-1/3A-2
RHG6	2A-2/2A-3	3A-1/3A-2
**WT bNAb Tx**		
RHEL	2A-1	3A-1
RHR6	2A-1/2A-2	3A-2
RHZ9	2A-1/2A-2	3A-2
RHCK*	2A-2	3A-1/3A-2
RHDL	2A-2/2A-3	3A-1
RHIV	2A-1	3A-1/3A-2
RHG4	2A-1/2A-3	3A-1
RHX4	2A-1/2A-2	3A-2
RHL9*	2A-1	3A-1/3A-2
RH51	2A-2/2A-3	3A-1/3A-2
**DEL bNAb Tx**		
RHRI	2A-2/2A-3	3A-1
RHFD*	2A-2/2A-3	3A-1
RHBG	2A-1/2A-2	3A-2
RHGC*	2A-2/2A-4	3A-2/3A-3
RHIL*	2A-1	3A-1/3A-2
RHFJ	2A-1	3A-1/3A-2
RHX0	2A-3	3A-1
RHH1	2A-1/2A-2	3A-2
RH41	2A-1	3A-1/3A-2
RHBC	2A-2	3A-1/3A-2

*uninfected animals
Tx, treatment; UnTx, untreated.

Supplementary Table 9. Antibodies used in flow cytometry and sorting experiments

Marker	Fluorochrome	Clone	Supplier
CCR7	AF700	150503	BD Biosciences
CD3	APC-Cy7	SP34-2	BD Biosciences
CD3	PE-CF594	SP34-2	BD Biosciences
CD4	AF700	OKT4	Biolegend
CD4	BUV661	SK3	BD Biosciences
CD8	BV570	RPA-T8	Biolegend
CD8	BV605	SK1	BD Biosciences
CD8	BV785	RPA-T8	Biolegend
CD14	AF700	M5E2	BD Biosciences
CD14	BV510	M5E2	Biolegend
CD16	BV510	3G8	Biolegend
CD16	BV711	3G8	Biolegend
CD16	BUV496	3G8	BD Biosciences
CD20	BUV737	2H7	BD Biosciences
CD20	BV510	2H7	Biolegend
CD20	Pacific Blue	2H7	Biolegend
CD20	PerCP/Cy5.5	2H7	Biolegend
CD28	ECD	CD28.2	Beckman Coulter
CD41a	BV711	HIP8	BD Biosciences
CD69	BV605	FN50	Biolegend
CD95	PECy5	DX2	BD Biosciences
CD107a	BV650	H4A3	Biolegend
CD282	PE	11G7	BD Biosciences
CXCR5	Biotin	MU5UBEE	eBioscience
CXCR5	PE	MU5UBEE	eBioscience
CXCR5	PECy7	MU5UBEE	eBioscience
HLA-DR	BV650	L243	Biolegend
HLA-DR	BV785	L243	Biolegend
HLA-DR	PE-Cy5.5	TU36	Life Technologies
Human kappa light chain	AF647	RM126	Novus Biologicals
Human lambda light chain	AF488	RM127	Novus Biologicals
IFNγ	FITC	B27	BD Biosciences
MIP-1β	PE	D21-1351	BD Biosciences
NHP CD45	FITC	D058-1283	BD Biosciences
NKG2A	APC	REA110	MACS Miltenyi Biotec
NKG2A	PECy7	Z199	Beckman Coulter
NKG2A	PE-Vio 770	REA110	MACS Miltenyi Biotec
PD-1	BV711	EH12.2H7	Biolegend
TNF	BV785	MAb11	Biolegend
