# Supplementary material for: Administration of anti-HIV-1 broadly neutralizing monoclonal antibodies with increased affinity to Fcγ receptors during acute SHIVAD8-EO infection
Source: Nat Commun. 2024 Aug 29;15:7461. doi: 10.1038/s41467-024-51848-y (PMC11358508; doi:10.1038/s41467-024-51848-y)
Supplement: Supplementary file 3 — Reporting Summary [file 41467_2024_51848_MOESM3_ESM.pdf]

nature portfolio

Corresponding author(s): Richard A. Koup

Last updated by author(s): Aug 2, 2024

Reporting Summary

Nature Portfolio wishes to improve the reproducibility of the work that we publish. This form provides structure for consistency and transparency in reporting. For further information on Nature Portfolio policies, see our Editorial Policies and the Editorial Policy Checklist.

Statistics

For all statistical analyses, confirm that the following items are present in the figure legend, table legend, main text, or Methods section.

n/a	Confirmed	
☐	☒	The exact sample size (*n*) for each experimental group/condition, given as a discrete number and unit of measurement
☐	☒	A statement on whether measurements were taken from distinct samples or whether the same sample was measured repeatedly
☐	☒	The statistical test(s) used AND whether they are one- or two-sided<br>*Only common tests should be described solely by name; describe more complex techniques in the Methods section.*
☒	☐	A description of all covariates tested
☐	☒	A description of any assumptions or corrections, such as tests of normality and adjustment for multiple comparisons
☐	☒	A full description of the statistical parameters including central tendency (e.g. means) or other basic estimates (e.g. regression coefficient) AND variation (e.g. standard deviation) or associated estimates of uncertainty (e.g. confidence intervals)
☐	☒	For null hypothesis testing, the test statistic (e.g. *F*, *t*, *r*) with confidence intervals, effect sizes, degrees of freedom and *P* value noted<br>*Give P values as exact values whenever suitable.*
☒	☐	For Bayesian analysis, information on the choice of priors and Markov chain Monte Carlo settings
☒	☐	For hierarchical and complex designs, identification of the appropriate level for tests and full reporting of outcomes
☐	☒	Estimates of effect sizes (e.g. Cohen's *d*, Pearson's *r*), indicating how they were calculated

Our web collection on statistics for biologists contains articles on many of the points above.

Software and code

Policy information about availability of computer code

Data collection	QuantStudio 12K Flex Software v.1.2.4 (Thermo Fisher Scientific)<br>QuantStudio Real Time PCR Software v1.4 (Thermo Fisher Scientific)<br>SoftMax Pro v.5.4.6 and v.7.1 (Molecular Devices, LLC)<br>SMRT Link v. 11.0.0.146107 (Pacific Biosciences)<br>Imaris v.9.9.0 and 10.1.0 (Bitplane)<br>BD FACS Diva Software (BD Biosciences)<br>Bcl2fastq v.2.20<br>xPONENT for MAGPIX v.4.2 (Luminex Corporation)
Data analysis	QuantStudio 12K Flex Software v.1.2.4 (Thermo Fisher Scientific)<br>QuantStudio Real Time PCR Software v1.4 (Thermo Fisher Scientific)<br>FileMaker Pro v20.2.1.60 and various v19 releases<br>Geneious Prime v.2020.0.5 (Biomatters Ltd.)<br>SoftMax Pro v.7.1 (Molecular Devices, LLC)<br>Imaris v.9.9.0 and 10.1.0 (Bitplane)<br>FlowJo v.9.9.6 and v.10.8.1 (BD Biosciences)<br>SPICE v.6.0 (Mario Roederer, VRC, NIAID, NIH)<br>xPONENT for MAGPIX v.4.2 (Luminex Corporation)<br>Prism v.9 (GraphPad Software, LLC)<br>For neutralization assay data analysis, interpolated percent neutralization values were determined using a custom-coded laboratory information management system.

The bioinformatics analysis pipeline developed for SHIVAD8-EO Env deep sequencing is available at https://github.com/niaid/UMI-pacbio-pipeline/releases; UMI-pacbio-pipeline v.1.0, lima v.2.5.1, vsearch v.2.21.1, Cutadapt v.4.1, minimap2 v.2.24, bcftools v.1.13, blastn v.2.9.0.

The bioinformatics analysis pipelines used for preprocessing and downstream analyses of the LN transcriptomic data are available at https://github.com/sekalylab/mRNASeq and https://github.com/sekalylab/joana, respectively; STAR aligner v.2.7.10a, HTSeq v.2.0.2, edgeR v.3.38.4, GSEA v4.1.0, MSigDB v.7.4, ComplexHeatmap v.2.12.1.

For manuscripts utilizing custom algorithms or software that are central to the research but not yet described in published literature, software must be made available to editors and reviewers. We strongly encourage code deposition in a community repository (e.g. GitHub). See the Nature Portfolio guidelines for submitting code & software for further information.

Data

Policy information about availability of data

All manuscripts must include a data availability statement. This statement should provide the following information, where applicable:

- Accession codes, unique identifiers, or web links for publicly available datasets
- A description of any restrictions on data availability
- For clinical datasets or third party data, please ensure that the statement adheres to our policy

The raw SHIVAD8-EO Env PacBio CCS sequencing data generated in this study have been deposited in the NCBI SRA database under the BioProject accession number PRJNA957445 (https://www.ncbi.nlm.nih.gov/bioproject/PRJNA957445/). The raw LN RNA sequencing data generated in this study have been deposited in the NCBI GEO database under the accession number GSE254837 (https://www.ncbi.nlm.nih.gov/geo/query/acc.cgi?acc=GSE254837). Source data are provided with this paper.

Research involving human participants, their data, or biological material

Policy information about studies with human participants or human data. See also policy information about sex, gender (identity/presentation), and sexual orientation and race, ethnicity and racism.

Reporting on sex and gender	No research involving human participants, their data, or biological material.
Reporting on race, ethnicity, or other socially relevant groupings	No research involving human participants, their data, or biological material.
Population characteristics	No research involving human participants, their data, or biological material.
Recruitment	No research involving human participants, their data, or biological material.
Ethics oversight	No research involving human participants, their data, or biological material.

Note that full information on the approval of the study protocol must also be provided in the manuscript.

Field-specific reporting

Please select the one below that is the best fit for your research. If you are not sure, read the appropriate sections before making your selection.

☒ Life sciences ☐ Behavioural & social sciences ☐ Ecological, evolutionary & environmental sciences

For a reference copy of the document with all sections, see nature.com/documents/nr-reporting-summary-flat.pdf

Life sciences study design

All studies must disclose on these points even when the disclosure is negative.

Sample size

We first used 6 naïve rhesus macaques in the WT bNAb-treated group, in which we hypothesized the following:
a) 100% animals will develop short-term viral control during the first few weeks of bNAb treatment;
b) 50% of the animals will develop long-term viral control after early treatment with the two-bNAb combination.
A sample size of 6 gave us >90% power to assess if bNAb treatment led to short-term viral control in all 6 animals, where short-term viral control was defined by plasma viral load <15 copies/ml by week 6 post-challenge.
We initiated the DEL bNAb-treated group with 6 monkeys after we observed that all 6 animals in the WT bNAb-treated group showed short-term viral control.
As controls for the treated animals in this study, we included 6 monkeys that were not treated with bNAbs.
After initiating 6 monkeys per group, we added 4 more monkeys in each group. Since plasma viremia did not segregate the animals into the two different batches (as depicted in Figure 1, B-E), viremic animals from both batches were grouped together for all subsequent analyses. This information was reported in the first Results section of the manuscript.

Data exclusions

Some SHIVAD8-EO-challenged monkeys never became infected (one untreated monkey, two WT bNAb-treated monkeys, and three DEL bNAb-treated monkeys), as detailed in the manuscript. Unless otherwise indicated in the manuscript, these uninfected monkeys were

excluded from further analyses.

Replication

For viral load analyses in plasma and cells, all assay runs included a standard curve and positive and negative control reaction wells. Per protocol, runs that failed to produce a satisfactory standard curve or acceptable values for control samples, as determined by comparison to extensive historical averages, were discarded and re-assay was performed with residual sample or replicate aliquots as available. All cell/tissue samples were assayed with 10 replicates and 2 “internal control spike wells” to monitor for potential reaction inhibition. Individual samples that had inhibited PCR reactions, as evidenced by unsatisfactory “Spike values”, were diluted and re-assayed. Plasma samples were assayed with 6 replicate reactions per specimen. Fc gamma receptor genotyping was performed in duplicate, pharmacokinetics ELISAs were performed using 4 dilutions per sample, with each dilution measured twice, and anti-drug antibody ELISAs were performed using 7 dilutions per sample, with each dilution measured once. For neutralization assays using plasma samples or bNAbs, dilution points for each sample or bNAb were run in duplicate. bNAbs were tested against SHIVAD8-EO in at least 5 experiments, with no more than 2-fold variability between them. All other experiments were performed once. Additional replicate experiments were not performed because the first round of experiments successfully produced quality data and additional experiments were not believed to affect the interpretations made from the first round of experiments.

Randomization

Monkeys were assigned to each group based on their FCGR2 and FCGR3 genotype for a balanced FCGR genotype representation across groups (Supplemental Table 8).

Blinding

This study was not blinded. Knowing the allocation of each monkey was critical to perform several assays such as the PK and ADA ELISAs and bNAb imaging.

Reporting for specific materials, systems and methods

We require information from authors about some types of materials, experimental systems and methods used in many studies. Here, indicate whether each material, system or method listed is relevant to your study. If you are not sure if a list item applies to your research, read the appropriate section before selecting a response.

Materials & experimental systems

n/a	Involved in the study
☐	☒ Antibodies
☐	☒ Eukaryotic cell lines
☒	☐ Palaeontology and archaeology
☐	☒ Animals and other organisms
☒	☐ Clinical data
☒	☐ Dual use research of concern
☒	☐ Plants

Methods

n/a	Involved in the study
☒	☐ ChIP-seq
☐	☒ Flow cytometry
☒	☐ MRI-based neuroimaging

Antibodies

Antibodies used

--> Antibodies for flow cytometry and sorting experiments:
Marker Fluorochrome Clone Supplier
CCR7 AF700 150503 BD Biosciences
CD3 APC-Cy7 SP34-2 BD Biosciences
CD3 PE-CF594 SP34-2 BD Biosciences
CD4 AF700 OKT4 Biolegend
CD4 BUV661 SK3 BD Biosciences
CD8 BV570 RPA-T8 Biolegend
CD8 BV605 SK1 BD Biosciences
CD8 BV785 RPA-T8 Biolegend
CD14 AF700 M5E2 BD Biosciences
CD14 BV510 M5E2 Biolegend
CD16 BV510 3G8 Biolegend
CD16 BV711 3G8 Biolegend
CD16 BUV496 3G8 BD Biosciences
CD20 BUV737 2H7 BD Biosciences
CD20 BV510 2H7 Biolegend
CD20 Pacific Blue 2H7 Biolegend
CD20 PerCP/Cy5.5 2H7 Biolegend
CD28 ECD CD28.2 Beckman Coulter
CD41a BV711 HIP8 BD Biosciences
CD69 BV605 FN50 Biolegend
CD95 PECy5 DX2 BD Biosciences
CD107a BV650 H4A3 Biolegend
CD282 PE 11G7 BD Biosciences
CXCR5 Biotin MU5UBEE eBioscience
CXCR5 PE MU5UBEE eBioscience
CXCR5 PECy7 MU5UBEE eBioscience
HLA-DR BV650 L243 Biolegend
HLA-DR BV785 L243 Biolegend

HLA-DR PE-Cy5.5 TU36 Life Technologies
Human kappa light chain AF647 RM126 Novus Biologicals
Human lambda light chain AF488 RM127 Novus Biologicals
IFNγ FITC B27 BD Biosciences
MIP-1β PE D21-1351 BD Biosciences
NHP CD45 FITC D058-1283 BD Biosciences
NKG2A APC REA110 MACS Miltenyi Biotec
NKG2A PECy7 Z199 Beckman Coulter
NKG2A PE-Vio 770 REA110 MACS Miltenyi Biotec
PD-1 BV711 EH12.2H7 Biolegend
TNF BV785 MAb11 Biolegend

--> Antibodies for ELISAs:
Antibody Supplier Cat #
Anti-Human IgG, Monkey ads-HRP Southern Biotech 2049-05
Anti-Monkey IgG-HRP Southern Biotech 4700-05
VRC07-523-LS lot# SKW381-140-05, harvest# B360-043 VRC/NIAID/NIH N/A
PGT121 lot# XJCL112217 VRC/NIAID/NIH N/A
VRC07-523-LS/DEL lot# 071918CL VRC/NIAID/NIH N/A
PGT121/DEL lot# 110618CL VRC/NIAID/NIH N/A
5C9 lot# 022119CL VRC/NIAID/NIH N/A
9E9 lot# 05-18-2016 VRC/NIAID/NIH N/A

--> Antibodies for imaging experiments:
Marker Fluorochrome Clone Supplier
CD3 N/A (unconjugated) F7.2.38 Dako
Anti-mouse IgG1a Alexa Fluor 594 N/A ThermoFisher Scientific
CD20 eFluor 660 L26 eBioscience
CD4 Alexa Fluor 700 polyclonal antibody (cat# FAB8165N) R&D Systems
CD20 N/A (unconjugated) L26 eBiosciences
Anti-Mouse IgG2a BV421 N/A Biolegend
Human kappa light chain AF647 RM126 Novus Biologicals
Human lambda light chain AF488 RM127 Novus Biologicals

--> Antibodies for infusions:
Antibody Supplier Cat #
VRC07-523-LS lot# SKW381-140-05, harvest# B360-043 and lot# 050819CL VRC/NIAID/NIH N/A
PGT121 lot# LW-638-04-01 and lot# XJCL112217 VRC/NIAID/NIH N/A
VRC07-523-LS/DEL lot# CL071918 and lot# 012220 VRC/NIAID/NIH N/A
PGT121/DEL lot# CL110618 and lot# 111318CL VRC/NIAID/NIH N/A
anti-CD8beta NIH Nonhuman Primate Reagent Resource PR-2557

Validation

All reagent antibodies obtained from commercial sources or the NIH Nonhuman Primate Reagent Resource were not validated further. Antibodies VRC07-523-LS, PGT121, VRC07-523-LS/DEL, and PGT121/DEL, which were produced at the VRC/NIAID/NIH, were validated in neutralization assays against the challenge virus SHIVAD8-EO. Antibodies 5C9 and 9E9, which were also produced at the VRC/NIAID/NIH, were validated by ELISA for binding to VRC07-523 and PGT121, respectively.

Eukaryotic cell lines

Policy information about cell lines and Sex and Gender in Research

Cell line source(s)

HeLa-derived TZM-bl cells (NIH AIDS Reagent Program, Cat#8129-442, RRID:CVCL_B478, sex: female) and HEK 293T cells (ATCC, Cat#CRL-3216, RRID:CVCL_0063, sex: female).

Authentication

None of the cell lines used were authenticated.

Mycoplasma contamination

Mycoplasma testing was performed biannually.

Commonly misidentified lines
(See ICLAC register)

None used.

Animals and other research organisms

Policy information about studies involving animals; ARRIVE guidelines recommended for reporting animal research, and Sex and Gender in Research

Laboratory animals

Male and female rhesus macaques (Macaca mulatta) of Indian origin and aged 2 to 4 years at the time of challenge were used in the study.

Wild animals

The study did not involve wild animals.

Reporting on sex

Both male and female rhesus macaques were used in the study. Sex-stratified analyses were not performed as the sex balance was not adequate and the study design was insufficient to draw meaningful conclusions.

Field-collected samples	The study did not involve samples collected from the field.
Ethics oversight	All animal procedures and experiments were conducted according to NIH regulations and standards on the humane care and use of laboratory animals as well as the Animal Care and Use Committees of the NIH Vaccine Research Center (VRC) and Bioqual, Inc. (Rockville, MD).

Note that full information on the approval of the study protocol must also be provided in the manuscript.

Plants

Seed stocks	N/A
Novel plant genotypes	N/A
Authentication	N/A

Flow Cytometry

Plots

Confirm that:

☒ The axis labels state the marker and fluorochrome used (e.g. CD4-FITC).

☒ The axis scales are clearly visible. Include numbers along axes only for bottom left plot of group (a 'group' is an analysis of identical markers).

☒ All plots are contour plots with outliers or pseudocolor plots.

☒ A numerical value for number of cells or percentage (with statistics) is provided.

Methodology

Sample preparation	Peripheral blood samples and lymph node (LN) biopsies were collected from monkeys at different timepoints. Peripheral blood mononuclear cells (PBMCs) and LN cells were isolated as described in our previous paper Dias et al., J Clin Invest, 2021 (PMID: 34623326) and stored in liquid nitrogen until further use. For cell sortings and other flow cytometry experiments, cryopreserved PBMCs and/or LN cells were thawed in RPMI-1640 medium supplemented with 10% fetal bovine serum, 2 mM L-glutamine, 100 µg/mL streptomycin, and 100 U/mL penicillin (all from Gibco). After washing, cells were either stained immediately (for cell sortings, immunophenotyping assays, and in vitro bNAb binding assays) or rested for one hour at 37 degrees/5% CO_2 in completed medium before proceeding to functional assays. Absolute cell counts were performed using fresh, whole monkey blood.
Instrument	For cell sortings: modified FACSAria III or modified FACSymphony S6 cell sorter equipped with 355-, 405- (FACSAriaIII) or 407- (FACSymphony S6), 488-, 532-, and 628-nm lasers (BD Biosciences). For all other flow cytometry experiments: modified BD FACSymphony equipped with 355-, 405-, 488-, 532- and 628-nm lasers (BD Biosciences).
Software	Flow cytometry data were analyzed using FlowJo v.9.9.6 and v.10.8.1 (BD Biosciences).
Cell population abundance	Several immune cell populations were sorted from monkey lymph node cells. Sorted samples had 81 to 5000 cells and their purity was determined by flow cytometry using the gating strategy used for sorting.
Gating strategy	Gating strategies and gate boundaries are provided in:<br>- Supplemental Figure 1B for absolute count of several immune cell populations in monkey whole blood.<br>- Supplemental Figure 5A to identify several immune cell populations in monkey lymph node cells (phenotypic assays).<br>- Supplemental Figure 6A to identify several immune cell populations in monkey lymph node cells (functional assays).<br>- Supplemental Figure 8 to sort several immune cell populations from monkey lymph node cells.

☒ Tick this box to confirm that a figure exemplifying the gating strategy is provided in the Supplementary Information.
